# Combinations

**Published:** 1882-07

**Authors:** J. B. Chandler


### COMBINATIONS.

J. B. CHANDLER.

The power of combined effort for the production of good or evil has been known to men probably as long as human beings have existed. History, sacred and profane, is but a continued chronicle of results from men associated for a purpose.

Good governments have been overthrown, and bad ones established, through the instrumentality of combinations with vicious leaders.

The people, restive under oppressive rulers, have combined and thrown off their thraldom. The weak become strong from combination, and the strong weak when their following is lost.

Useless then would it be to attempt to argue against men associating together, for all arguments will be met with the irrefutable assertion of the immense power for doing good which is thus obtained.

The great question of the present is the harmonizing of labor and capital. Not that of necessity there exists any antagonism between them, because here, one is worthless without the other, but the immense immigration of workmen from Europe, who bring with them habits of thought engendered in the crowded channels of labor in the old countries, has entailed upon us the trade combinations and their power of evil in disturbing the natural channels of improvements and trade.

There is no doubt of the immense power for good which associations of trade might possess, such as the righting of real wrongs, the insurance of medical attendance and family support to sick and crippled members, the fund for burial, or life insurance for benefit of widows and orphans. All these, and perhaps more might be enumerated, are legitimate and praiseworthy objects for such associations, and all these can be carried out by the members themselves, with special rules and a secretary to obey orders from the society. No autocrat need be elected to whose *ipse dixit* members must bow and yield implicit obedience—whose power is to be perpetuated by stirring up dissensions between employer and employe—who, because a strife exists in Pittsburgh, will order another in Chicago where all may be harmony between the mill-owner and his men—and thus men be thrown out of employment to live in idleness off a fund contributed from the surplus of wages which are claimed to be inadequate for the necessities of life.

The fixing of the price of production of any manufactured article is extremely difficult. The attempt to bring all advantages of position to one level is absurd. The cost of living in some sections of the country is double that in others. The workman who gets two dollars a day in one place may put away money ; whilst in another he who, with an equal family, receives three dollars, will find it hard to get along.

The abolishment of piece-work and the enforcement of a fixed per diem must inevitably deprive the skilled and industrious workman of the advantage he possesses over the stupid and lazy. When trade is brisk, if the employer must pay the poor workman more than he is worth, he cannot give the skilled man any advantage, except in the preference of employing him.

Out of combinations in other quarters arise some of the real evils upon which advocates of trade-unions base strong arguments in their favor. One of the worst is the combination of speculators in grain and provisions, who, checking the supply, enforce advance in prices before the demand is met. Thus the channels of trade are disturbed as by a famine, and the purchase value of a dollar, instead of being fixed by a labor standard, is temporarily at the mercy of the "corner" combinations. Then, whilst contracts are being filled for building, iron or other manufactures, based upon existing prices of production, comes a general demand for higher wages, and the country is threatened with strikes and riots. And those who claim they cannot live on wages received produce a fund,* claimed to be inexhaustible, hoarded from the surplus of those wages and rush into idleness, and too often dissipation and crime. The corner speculations in the necessaries of life are within the reach of the law, and such traffic should be put down effectually. But to reach the abuses of trade-union strikes, until by some overt act the law is broken, there is needed special dealing with the workmen themselves. Many are intelligent enough to understand, when the facts are laid before them, the immense pecuniary loss to themselves directly, and reflectively also, from the loss to the country by the idleness of its citizens and the consequent patronage of foreign workmen.

In a country like ours, filled with instances of men rising from the lowest to the highest walks of life—from poverty to riches—the workman of to-day must remember that he may be the employer of to-morrow; and in studying, as he should do, to fit himself for that position he should be able to see where lies the point at which both interests meet.

There is no disposition (certainly no general one) in this country to oppress the laboring classes. In no country in the world do they receive higher wages or live better. There is no need for strikes, then. A real grievance, which cannot be abated by manly arbitration with the employer, would soon succumb to public opinion, which is always with the oppressed.

"Whilst the pot is kept boiling the scum is always on top." Let all the questions of prices and time settle themselves in the natural way of supply and demand and the honest and frugal mechanic will never be out of employment or want for means to support his family in comfort far beyond any of the workmen of the old countries.

The fund now devoted to supporting idle and traveling officers and promulgating strikes and laziness will be well spent in elymosynary aid to worthy associates and their families in sickness and distress.

The great underlying principle of our form of government is the ability of people to govern themselves, the willingness of each to sacrifice some personal liberty when it becomes necessary for the general good, as instanced in obeying laws which curtail privileges which would be only abused by the vicious and intemperate.

But there is a point at which these concessions must stop, otherwise we abandon our freedom altogether. How, then, are we to reach the abuses of the press when men who, driven from their own countries for treason, combine here to teach through that medium treason to all government, murder to all rulers ?

These and other kindred abuses can only be corrected by sound public opinion or arbitrary power of government. Let us adhere to the first as long as possible. Let the blatant disorganizer feel the weight of public censure. Frown down the orator whose capital of gab consists in preaching discontent. Give the people to know that riches and high official position do not insure happiness. And more especially let it be known that no one, rich or poor, can escape the horrors which follow in the track of anarchy. And anarchy can but result from the realization of the schemes of some combinations in existence now, both in Europe and in this country.

---

This edition of the BISTOURY is delayed two weeks to give the editor an opportunity for his vacation in the woods—a yearly occurrence with him, in the month of June.

---

*NOTE.—A special despatch from Pittsburgh, says :—" We have money enough to support the 30,000 strikers for over three months," said a prominent officer of the Amalgamated Association, "and can raise as much more in forty-eight hours."

